# Peripheral Inflammatory Markers and Antioxidant Response during the Post-Acute and Chronic Phase after Severe Traumatic Brain Injury

**DOI:** 10.3389/fneur.2016.00189

**Published:** 2016-11-02

**Authors:** Federico Licastro, Silvana Hrelia, Elisa Porcellini, Marco Malaguti, Cristina Di Stefano, Cristina Angeloni, Ilaria Carbone, Laura Simoncini, Roberto Piperno

**Affiliations:** ^1^Department of Experimental, Diagnostic and Specialty Medicine, University of Bologna, Bologna, Italy; ^2^Department for Life Quality Studies, University of Bologna, Rimini, Italy; ^3^Neurorehabilitation Unit, Emergency Department, Maggiore Hospital, Bologna, Italy

**Keywords:** traumatic brain injury, inflammation, oxidative stress, cytokines plasma levels, follow-up, cognitive outcome

## Abstract

Traumatic brain injury (TBI) is a mechanical insult to the brain caused by external forces and associated with inflammation and oxidative stress. The patients may show different profiles of neurological recovery and a combination of oxidative damage and inflammatory processes can affect their courses. It is known that an overexpression of cytokines can be seen in peripheral blood in the early hours/days after the injury, but little is known about the weeks and months encompassing the post-acute and chronic phases. In addition, no information is available about the antioxidant responses mediated by the major enzymes that regulate reactive oxygen species levels: superoxide dismutase, catalase, peroxidases, and GSH-related enzymes. This study investigates the 6-month trends of inflammatory markers and antioxidant responses in 22 severe TBI patients with prolonged disorders of consciousness, consecutively recruited in a dedicated neurorehabilitation facility. Patients with a high degree of neurological impairment often show an uncertain outcome. In addition, the profiles of plasma activities were related to the neurological recovery after 12 months. Venous peripheral blood samples were taken blindly as soon as clinical signs and laboratory markers confirmed the absence of infections, 3 and 6 months later. The clinical and neuropsychological assessment continued up to 12 months. Nineteen patients completed the follow-up. In the chronic phase, persistent high plasma levels of cytokines can interfere with cognitive functioning and higher post-acute levels of cytokines [interferon (IFN)-γ, tumor necrosis factor (TNF)-α, IL1b, IL6] are associated with poorer cognitive recoveries 12 months later. Moreover, higher IFN-γ, higher TNF-α, and lower glutathione peroxidase activity are associated with greater disability. The results add evidence of persistent inflammatory response, provide information about long-term imbalance of antioxidant activity, and suggest that the over-production of cytokines and the alteration of the redox homeostasis in the post-acute phase might adversely affect the neurological and functional recovery. Inflammatory and antioxidant activity markers might offer a feasible way to highlight some of the processes opposing recovery after a severe TBI.

## Introduction

Traumatic brain injury (TBI) is a mechanical insult to the brain caused by external forces resulting in temporary or permanent neurological deficits. TBI is a disorder with major health impact, since it may often result in lifelong impairments of physical, cognitive, and psychosocial functioning (1). Moreover, TBI is one of the leading causes of disability in children and young adults.

After a TBI, the brain damages occur in two distinct phases: the primary injury is caused immediately by the mechanical forces acting on the skull and the brain and the secondary injury consists in a cascade of events sustained by other mechanisms, such as ischemia, hypoxemia, and raised intracranial pressure. The secondary injury occurs progressively in minutes/hours after the primary injury and the resulting brain damages are exacerbated by oxidative stress, inflammation, and excitotoxicity (2).

Recent reports suggest that inflammation is associated with TBI (3, 4), and cerebral inflammatory responses appear to begin within minutes after TBI (5). In addition, high levels of inflammatory cytokines, such as interleukin (IL)-1β, IL-2, IL-4, IL-6, IL-8, IL-10, tumor necrosis factor (TNF)-α and interferon (IFN)-γ, have been reported in human postmortem brain tissue (5).

Oxidative stress occurs when the brain antioxidant mechanisms are overcome by increasing levels of reactive oxygen species (ROS) and reactive nitrogen species (RNS) (6). The oxidative stress plays a complex role in the mechanisms of damage after TBI (6, 7). The brain consumes about 20–30% of inspired oxygen, and contains high levels of polyunsaturated fatty acids and redox transition metals, and, therefore, is a vulnerable target for ROS attack (8). Brian intense metabolic activity, extensive production of ROS, reduced antioxidant capacity, and low levels of repair mechanisms in neurons may influence brain high susceptibility to oxidative stress (9). The abnormal ROS production downregulates tight junction proteins and activates matrix metalloproteinases (MMPs) that contribute to impair the blood–brain barrier (BBB) permeability.

Brain cells may use different types of antioxidants, including enzymes and low-molecular weight antioxidants. Superoxide dismutase (SOD), catalase (CAT), peroxidases, and GSH-related enzymes are the major enzymes that regulate ROS levels. The ROS are able to directly induce the synthesis and release of inflammatory cytokines, such as IL-1β and TNF-α (6).

These events are not confined to the first acute period after TBI injury. Pro-inflammatory molecules are released by activated microglia after TBI (10), and there is still evidence of increased microglial activity years after the brain traumatic event (11–13).

The presence of the allele 4 of the apolipoprotein E (APOE) gene, a known genetic risk factor for Alzheimer’s disease (AD), affects the progression of neurodegenerative processes in TBI patients (14) and is associated with increased neurologic complications (15).

Severe TBI has various sequelae with a wide range of severity. Similar injuries may greatly differ in clinical presentation and long-term outcome. After a severe TBI, people have been considered “survivors” facing permanent deficits, but the clinical evidence suggests that the TBI is not a pure traumatic event (16), since patients show different time and rate of recovery, or may result in progressive clinical deterioration.

Progressive white matter deterioration can persist for 1–2 years (17–21) or even for longer time intervals (22, 23).

The cognitive functioning after 1 year (24) or after 2–5 years (25) in 30% of the patients may worsen, and an increased incidence of AD and Parkinson’s disease has been associated with TBI (26).

The mechanisms underlying the outcome and history of functioning after severe TBI are complex and neuroinflammation and neurodegeneration may have a prominent role, as well as neuroplasticity and rewiring. The mechanisms underlying the neurodegeneration after a TBI are still not completely elucidated, but they might include a combination of oxidative damage and inflammatory processes.

Several studies have addressed the question whether the plasma inflammatory markers may offer clinically valuable information after TBI. Most studies describe increased serum levels of cytokines (mainly IL-6, IL-8, IL-10, and TNF-α) in serum shortly after the injury, i.e., hours to days (up to 5 days) (27–35). A small number of studies have investigated inflammatory factors during slightly longer periods such as 7 days (36, 37), 14 days (38), or 22 days (39). Only one study (40) has reported elevated serum levels of IL-1β, IL-6, IL-8, IL-10, and TNF-α in TBI patients’ blood samples taken during the subacute and the chronic phase (3–6 months) after the injury.

The present investigation explores the hypothesis that persistently high inflammatory responses may be associated with a slow or poor cognitive recovery and increased odds of late worsening. To validate the above notion 6-month trends of oxidative stress and inflammatory factors in the post-acute and chronic phases after a severe TBI have been investigated. Moreover, plasma profiles of oxidative stress-related enzymes and inflammatory makers have been related to the cognitive performances and recovery attained 12 months after TBI.

## Materials and Methods

### Subjects

Twenty-two patients with diagnosis of coma, vegetative state (SV), or minimally conscious state (MCS) were consecutively enrolled after they were transferred from the intensive care unit (ICU) to the neurorehabilitation unit of the Emergency Department, at the Maggiore Hospital in Bologna.

This study was approved by the Independent Ethics Committee of Bologna and was carried out according to the Helsinki Declaration (cod. CE 11076/2012), and a proxy consent was signed by a representative of each patient.

Patients with neurodegenerative diseases, history of clinical depression, other psychiatric disorders, and autoimmune and/or severe metabolic disorders were not included. Patients with previous motor or cognitive disabilities were also not included. Before the TBI, all subjects were able to carry out all usual duties and activities.

Structural damages along with secondary clinical and surgical complications of the neurological trauma were carefully recorded.

All patients (age range 18–65 years) suffered from a severe TBI [initial Glasgow Coma Score (GCS) ≤8 (41)] followed by a prolonged disorder of consciousness (DOC), resulting in level of cognitive functioning (LCF) <4 (42) and disability rating scale (DRS) >17 (43) at the recruitment time (between 15 and 40 days, median 28 days).

### Plasma Samples Collection

Venous peripheral blood samples were collected from patients into heparinized tubes and labeled by numeric code. Within 1 h, the blood samples were centrifuged at 1500 *g*, for 15 min at 14°C to obtain the plasma and buffy coat from each sample.

The plasma samples from TBI patients were taken for the first time (T0) as soon as the clinical signs and the laboratory markers (procalcitonin and leukocyte count) confirmed the absence of infections, between 15 and 66 days after the TBI (mean ± SEM: 32.4 ± 3.2 days). This time corresponds to the post-acute phase. Oxidative stress parameters and cytokine plasma levels were then evaluated two more times, always in absence of clinical and/or laboratory signs of infections: at T3, 3 months after T0 (104–176 days after the TBI, mean ± SEM: 128.1 ± 3.9) corresponding to a late post-acute/early chronic phase, and at T6, 6 months after T0 (193–296 days after the TBI, mean ± SEM: 224.7 ± 5.6) corresponding to the chronic phase.

No patient was taking any anti-inflammatory medication when the plasma samples were withdrawn. Samples processing and scoring were performed blind at the end of the study. Information about clinical status, biochemical data, and immunological data were linked to the code number and statistically analyzed.

### Chemicals and Reagents

NADPH, dimethyl sulfoxide (DMSO), 1-chloro-2,4-dinitrobenzene (CDNB), 5,5′-dithiobis(2-nitrobenzoic) acid (DTNB), reduced glutathione (GSH), oxidized glutathione (GSSG), 2-(4-iodophenyl)-3-(4-nitrophenyl)-5-(2,4-disulfophenyl)-2H-tetrazolium, monosodium salt (WST-1), xanthine oxidase (XO), and all other chemicals of the highest analytical grade were purchased from Sigma Chemical Co. (St. Louis, MO, USA).

### Enzymatic Activity Assays

Glutathione reductase (GR) activity was measured, as reported previously (44). Briefly, 30 μl of plasma was added to 970 μl of reaction mix (100 mM phosphate buffer, pH 7.5, containing 1 mM EDTA, 2 mM NADPH, and 2 mM GSSG). The decrease in absorbance at 340 nm was monitored spectrophotometrically for 1 min at 25°C. GR activity was expressed as units per milliliter. One unit of enzyme activity is defined as the amount of enzyme that causes the oxidation of 1.0 μmol of NADPH at 25°C at pH 7.5.

Catalase activity from plasma samples was determined according to the method of Johansson and Borg (45). The method is based on the enzyme reaction with methanol in the presence of an optimal concentration of hydrogen peroxide. The formaldehyde produced is measured spectrophotometrically with 4-amino-3-hydrazino-5-mercapto-1,2,4-triazole (Purpald) as a chromogen. CAT activity was expressed as nanomoles per minute per milliliter.

Superoxide dismutase activity was measured according to the method of Peskin and Winterbourn (46). This method allows SOD assessment by using a highly water-soluble tetrazolium salt, WST-1 that produces a water-soluble formazan dye upon reduction with a superoxide anion. The rate of the reduction with superoxide anion is linearly related to the XO activity and is inhibited by SOD. Therefore, the SOD activity was detected by a colorimetric method at 450 nm. Values obtained for each sample were compared to the concentration–response curve of standard SOD solutions and were expressed as units per milliliter. One unit of enzyme activity is defined as the amount of enzyme that inhibits the reduction of WST-1 by 50% in a coupled system with XO at pH 7.8 at 37°C.

Glutathione peroxidase (GPx) activity was spectrophotometrically measured, as described previously (47). The method was based on the reduction of GSSG coupled with the oxidation of NADPH. The decrease in absorbance at 340 nm was spectrophotometrically monitored at 25°C. GPx activity was expressed as units per milliliter. One unit of GPx activity was defined as the amount of enzyme that catalyzes the reduction of 1 μmol of NADPH per minute.

Total plasma antioxidant activity (TEAA) was measured, as reported previously (48). The method was based on the ability of the antioxidant molecules in the sample to reduce the radical cation of ABTS, determined by the decolorization of ABTS^+^, and measured as quenching of absorbance at 740 nm. Values obtained for each sample were compared to the concentration–response curve of a standard Trolox solution and expressed as millimole of Trolox equivalents (TE).

### Cytokine Assessment

Interleukin-1β, IL-6, IFN-γ, and TNF-α plasma levels were assessed by Bio-Plex Pro Human Cytokine 4-Plex Assay (Bio-Rad Laboratories, Hercules, CA, USA). Assays were performed following the manufacturer’s instructions (Multi beads assay BioPlex, BioRad), and cytokines levels were calculated according to the Bio-Plex Cytokine software.

### SNP Detection

Genomic DNA was extracted from peripheral blood leukocytes, as described previously (49).

Apolipoprotein E genotyping for the ϵ4 allele (rs429358, rs7412) was performed by TaqMan^®^ SNP genotyping assays (Applied Biosystems, Foster City, CA, USA), using a CFX96 BioRad Real-Time cycler and according the manufacturer’s instructions.

### Clinical and Cognitive Assessment

Functioning and disability were assessed by functional independence measure (FIM) (50) and DRS (43) every month up to 12 months.

Three months after TBI (T3), as soon as some communication skill was restored, and later every 3 months up to 1 year (T6, T9, and T12), the subjects were prospectively assessed by a structured neuropsychological battery specifically chosen to evaluate the cognitive functions after TBI (51).

The neuropsychological protocol included 16 tests administered according to validated procedures. The following domains were assessed: global cognitive functioning [Mini-Mental State Examination – MMSE (52)]; executive functioning [Tower of London (53), Wisconsin Card Sorting Test – WCST (54), letters and semantic fluency (55), behavioral assessment of the dysexecutive syndrome – BADS (56), trail making test – TMT (57), colored progressive matrix – CPM (58), Block design – WAIS (59, 60), Paced Auditory Serial Addition Task – PASAT (61)]; memory [Rey Auditory Verbal Learning Test (62), Episodic Memory Test (63), and Rey–Osterrieth Complex Figure Test (64)]; and attention [Allertness, Go/nogo, and Incompatibility from Test of Attentional Performance (65)].

In order to compare the results of different tools, the raw score of each neuropsychological test was converted into a standardized *z*-score. The *z*-scores were calculated by the mean scores and the SD of the normative values from a control population (25). Negative *z*-scores indicate a performance below the average of healthy subjects, whereas a *z*-score greater than 0 indicates an above-average performance.

A synthetic index (SI) of each cognitive function (66) for individual patient was then calculated. SI was expressed as the average of the *z*-scores of all the tests exploring the same cognitive domain, and an overall cognitive performance index (CPI) as the general average of all the *z*-scores.

A CPI higher than −1 meant an overall cognitive performance within or near the limits of normality (less than 1 SD), whereas a CPI lower than −1 meant an overall cognitive performance more than 1 SD below the lower limit of normality.

The CPI was preferred as the main outcome measure because it is assumed to be sensitive also to later worsening of the cognitive functioning. According to the CPI score 12 months after the TBI (T12), the cohort of patients has been divided in two groups with different cognitive outcome: the first group showing a T12-CPI higher than −1 was classified as good recovery, while the group with T12-CPI lower than −1 was classified as slow to recover (SR).

### Follow-up Analysis

The clinical and cognitive assessment continued up to 12 months, while the phenotypical variables were assessed at three different time: T0: baseline, T3: after 3 months, and T6: after 6 months.

Among the 22 subjects enrolled in the study, 1 patient deceased (007m12), 1 refused to continue in the study (004f12), and 1 was transferred to another hospital after 6 months (014m13). Nineteen patients completed the clinical and cognitive follow-up of 12 months.

### Statistical Analysis

The clinical, cognitive, and biomarkers results were reported as mean ± SEM. The cognitive outcome measures (T12-CPI) were dichotomized into CPI ≥ −1 and CPI < −1. Analysis of variance (ANOVA) was used to compare means as appropriate.

Two-way ANOVA (type I sequential Sum of Squares) and Tukey HSD *post hoc* correction were applied to test a design with factors being cognitive outcome and time point of the follow-up.

Linear models fitting and regression lines were used to explore predictors of the DRS.

Pearson’s product–moment correlation was also applied to correlate plasma levels to cognitive performances in time.

The statistical calculations were conducted using the Statistical Package for the Social Sciences (version 20.0; SPSS Inc., Chicago, IL, USA) and the R open source software (version 3.2.3; R Foundation for Statistical Computing). Significance was defined as *p* ≤ 0.05.

## Results

Patient’s demographic and clinical features are described in Table 1. Five patients were female. Age and education along with the cause of brain injury are detailed for each subject. The patient’s age was 38.0 ± 3.09 (mean ± SEM) years, while the years of education were 11.4 ± 0.69 (mean ± SEM).

**Table 1:** **Clinical and demographic data of TBI subjects**.

Code	Gender	Age	Education (years)	Cause of TBI	Diffuse injury	Lesions (CT scans)	Decompressive craniectomy	Skeletal trauma
001f12	F	47	13	Road accident	Y	Right frontal	Y	Y
002m12	M	61	17	Road accident	Y	Right frontal, bilateral temporal	N	N
003f12	F	53	13	Road accident	Y	Left fronto-temporo-parietal	Y	N
004f12	F	19	13	Road accident	Y	Right fronto-temporo-parietal	Y	Y
005f12	F	20	13	Road accident	Y	SAH, right mesencephalon	N	N
006m12	M	19	13	Road accident	Y	None	N	Y
007m12	M	54	NA	Road accident	Y	Left occipital-temporal	N	Y
008f12	F	43	13	Road accident	Y	Left frontal	N	N
009m12	M	26	17	Road accident	Y	Right external and internal capsule, cerebral peduncle, thalamus. Bilateral temporal	N	N
010m12	M	38	10	Road accident	Y	SAH. Right Cerebellum. Posterior left mesencephalon. Basal ganglia. Right mesial temporal. Bilateral fronto-temporal	N	N
011m12	M	25	11	Accident at work	Y	None	Y	N
012m13	M	30	8	Road accident	Y	Right fronto-temporal	N	Y
013m13	M	29	9	Road accident	Y	None	Y	Y
014m13	M	45	13	Road accident	Y	SAH. Fronto-temporal bilateral	N	N
015m13	M	27	10	Road accident	Y	SAH. Left frontal	Y	Y
016m13	M	20	13	Road accident	Y	None	N	Y
017m13	M	56	8	Road accident	Y	Left fronto-parietal, left occipital	N	Y
018m13	M	64	5	Fall	Y	Left frontal	N	Y
019m13	M	29	8	Road accident	Y	SAH. Bilateral fronto-parietal and temporal-polar. Mesencephalon	Y	Y
020m13	M	50	13	Road accident	Y	Left frontal-polar, fronto-parietal, and insula	N	Y
021m13	M	46	8	Road accident	Y	Right frontal, right temporal-polar, and internal capsule	N	Y
022m13	M	35	NA	Road accident	Y	None	Y	Y

*F, female; M, male; Y, yes; N, no; NA, not applicable*.

Brain computed tomography (CT) scans results are also reported. Localized brain lesions were not seen in five patients, five patients had subarachnoid hemorrhage (SAH), and three patients had also mesencephalic damages.

At the baseline, approximately 30 days after the TBI, all patients were still affected by a severe DOC, with a DRS score ranging from 18 to 26 (mean ± SEM: 22.3 ± 0.45). All patients had a FIM score of 18 (completely dependent on every functional activity) and were fed only by nasogastric tube or percutaneous gastrostomy with standard commercial products for enteral nutrition.

After 12 months, 2 patients (12m13 and 22m13) were still minimally conscious, while 17 patients regained consciousness: the outcome ranging from severe disability to good outcome. At T12, the FIM score was 88.25 ± 9.47 (mean ± SEM) with 6 patients who reached the highest scores (120–126) and complete independence in daily life activities; the DRS score was 7.3 ± 1.64 (mean ± SEM).

Between 3 and 9 months after the first assessment, most patients reached their best recovery in terms of dependence and disability (respectively, FIM and DRS scores).

**Figure 1:**
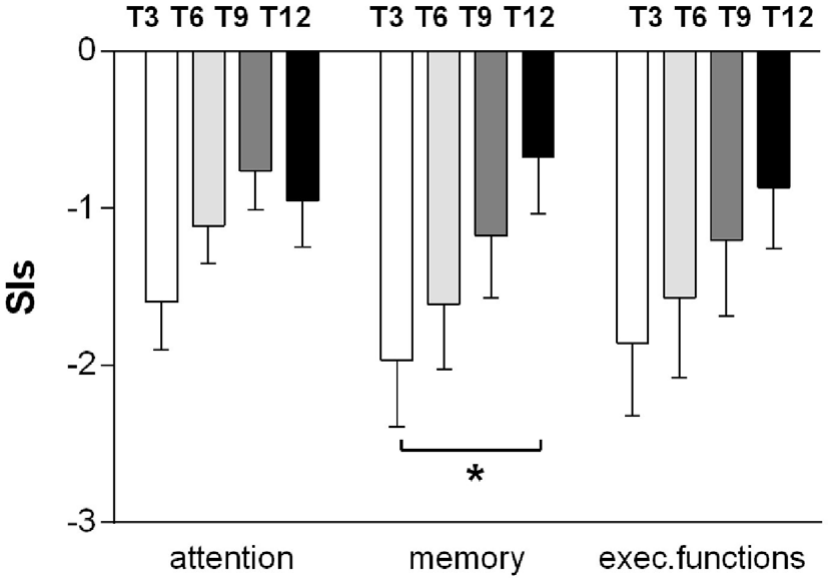
**Improvements in memory, attention, and executive functioning during the 12-month follow-up**. The results for the domains at every time point (T3, T6, T9, and T12) are reported as mean ± SEM of the synthetic index, expressed as the average of the *z*-scores of all the tests exploring the same cognitive domain. Measures were obtained from the 15 subjects who were able to perform the entire neuropsychological battery (*t*-test T3–T12. *p < 0.05).

The average cognitive functioning gradually improved during the 12-month follow-up, without sharp differences between the three domains (memory, attention, and executive functioning) as assessed by the SIs (Figure 1). The largest gain was for memory, with significant changes between T3 and T12 (*p* = 0.016), while attention lately worsens (at T12), although the differences did not reach statistical significance (Figure 1). The average CPI regularly improved from −1.807 at T3 up to −0.811 at T12.

Oxidative stress parameters and cytokine plasma levels were evaluated in TBI patients at three time points: at the enrollment in the study (T0) corresponding to a post-acute phase, after 3 months (T3), corresponding to a late post-acute/early chronic phase and 6 months (T6) after T0, corresponding to the chronic phase.

**Figure 2:**
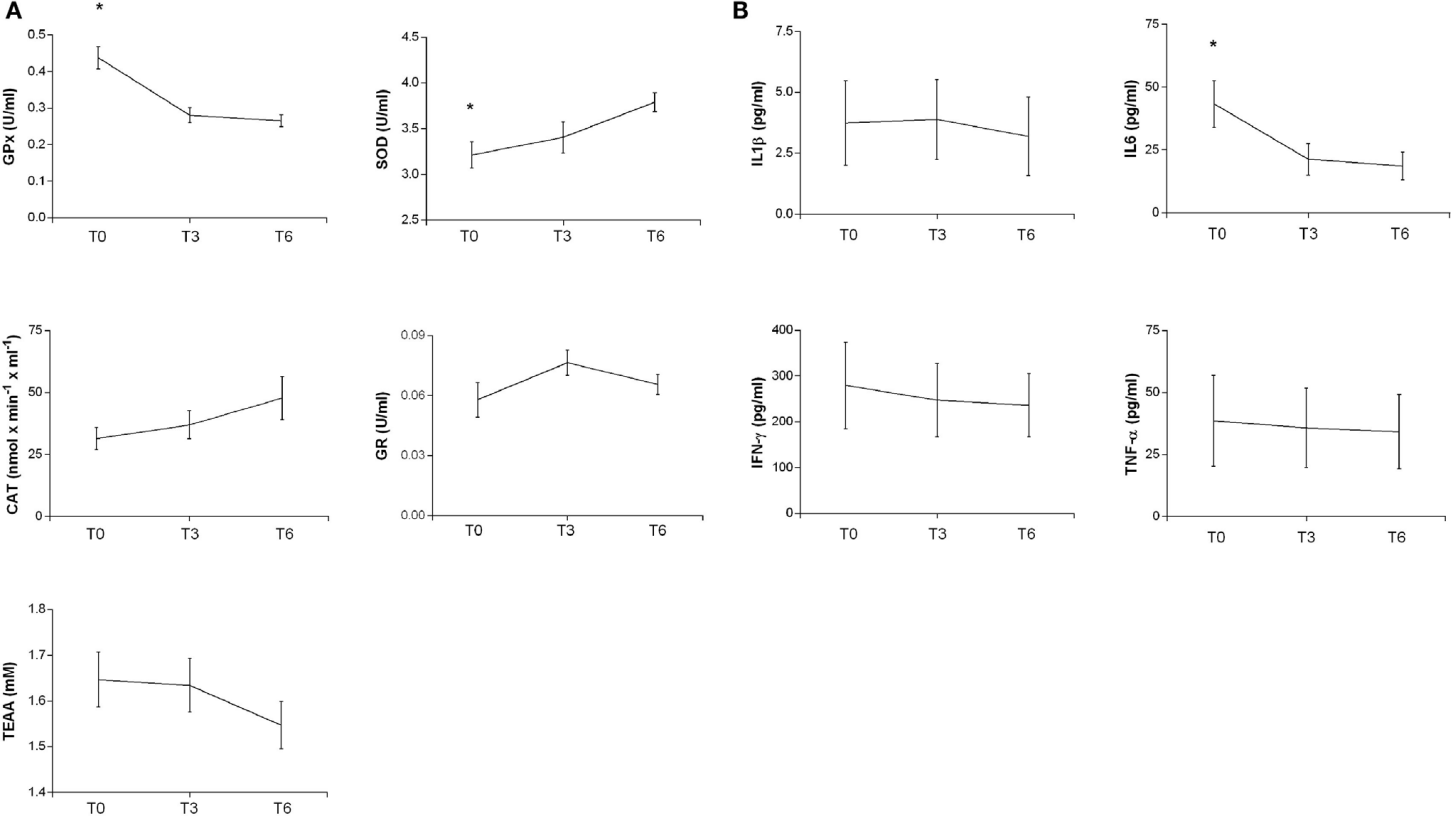
**Plasma antioxidant enzyme activities (A) and cytokine levels (B) in patients at different times after TBI**. SOD, CAT, GR, GPx activities, and TEAA **(A)** and IL-1b, IL6, IFN-γ, and TNF-α **(B)** were measured in plasma samples at different times as reported in Section “Materials and Methods.” The activities at T0 and after 3 and 6 months are reported. Statistical analysis was performed by one-way ANOVA followed by Tukey HSD post test (*p < 0.05).

In the post-acute phase (T0), GPx and IL6 levels were significantly higher than during the chronic phase (T6) (ANOVA with *post hoc* test Tukey HSD, *p* = 0.0000285 and *p* = 0.05000, respectively); the SOD levels instead were significantly lower (*p* = 0.0189674) (Figure 2). Linear regression analyses for SOD (intercept = 3.18357, slope = 0.09513, *p* = 0.007182), GPx (intercept = 0.416538, slope = −0.029617, *p* = 0.000219), and IL6 (intercept = 38.852, slope = −3.883, *p* = 0.03138) showed significant time trends during the 6-month follow-up. On the contrary, CAT, GR, TAA, IL1β, IFN-γ, and TNF-α did not show significant changes from T0 to T6.

**Figure 3:**
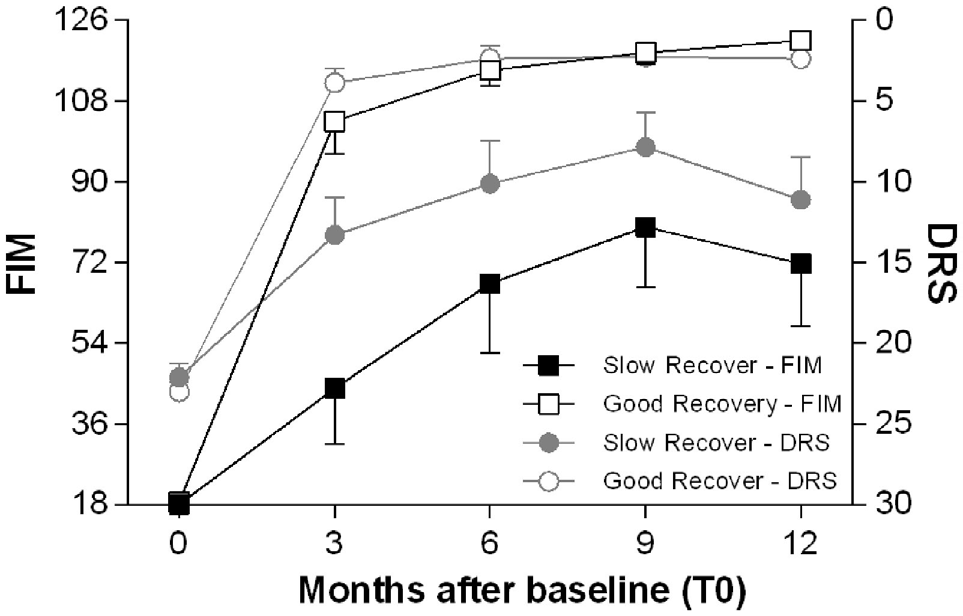
**Time course of functional recovery**. Average clinical measures of disability (DRS, right *y*-axis, range 0–30: worst score 30) and functional independence (FIM, left *y*-axis, range 18–126: worst score 18) at the enrollment (T0) and after 3, 6, 9, and 12 months. The patients are divided in two groups, good recovery (GR) and slow to recover (SR) according to the cognitive performance index (CPI) as described in Section “Materials and Methods.” The GR patients achieve most of the gains in DRS and FIM scores within 3 months after the TBI, while the SR patients reach later a plateau, approximately 9 months after the TBI.

TBI patients were then divided into two groups, according to the cognitive recovery attained after 12 months: good recovery (GR) (CPI > −1) and SR (CPI < −1). The two groups of patients differed for several aspects and also for the time of recovery: the GR group achieved earlier, approximately 3 months after TBI, a plateau of improvement in disability and dependence (as measured by DRS and FIM scores); the SR group reached their plateau, approximately 9 months after the TBI (Figure 3).

**Figure 4:**
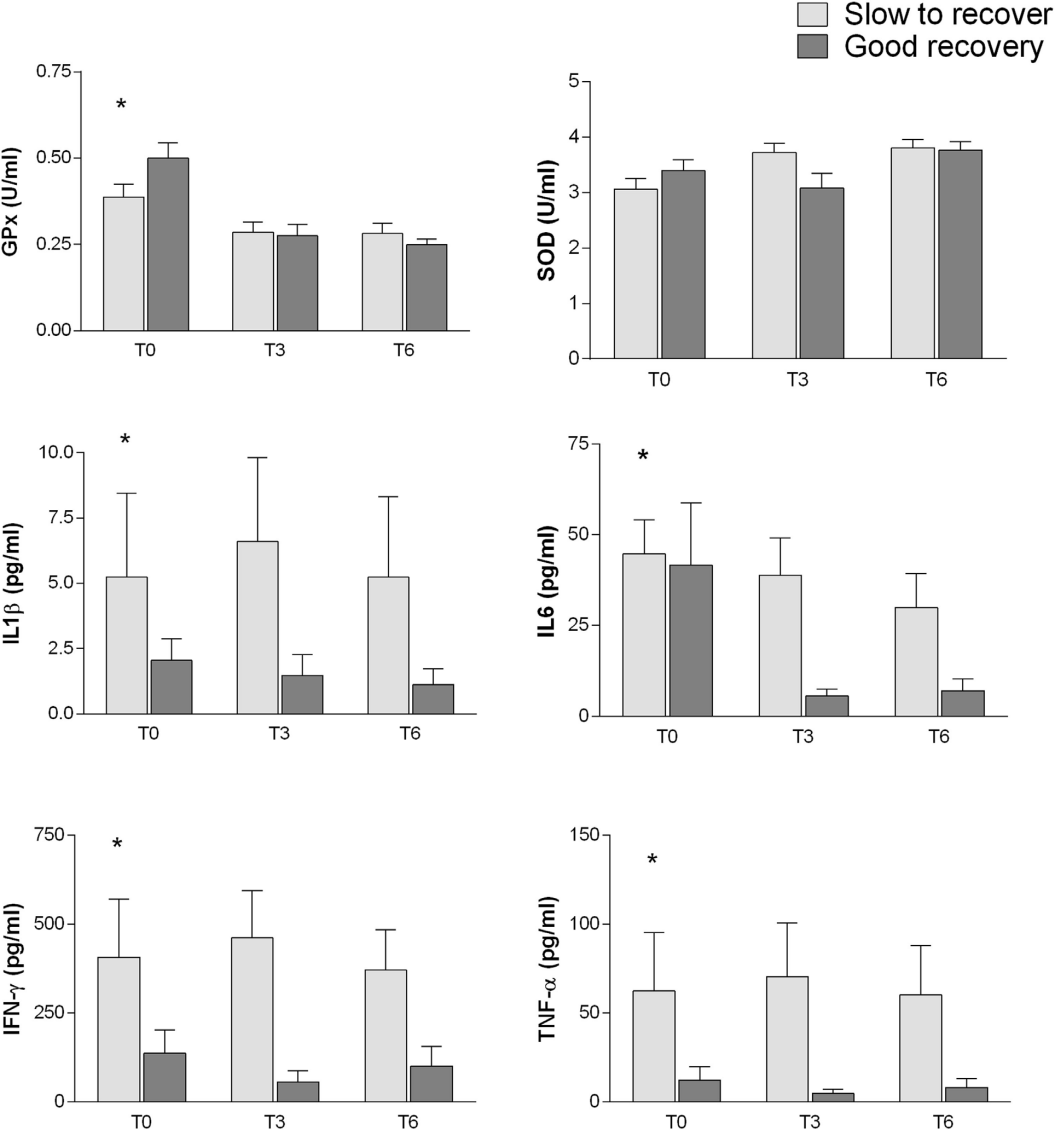
**Association between inflammatory markers and oxidative stress-related enzymes with different outcome (SR and GR)**. Analysis performed by two-way between subjects ANOVA (type I sequential Sum of Squares) and Tukey HSD *post hoc* correction; factors are cognitive outcome and time point of the follow-up. Higher plasma levels of IFN-γ (*p* = 0.017), TNF-α (*p* = 0.0033), IL6 (*p* = 0.0170), and IL1β (*p* = 0.0353) are related to poorer cognitive outcome. Plasma levels of GPx (*p* = 2.63e−06), SOD (*p* = 0.0179), and IL6 (*p* = 0.0342) change with time from injury.

The inflammatory markers and the oxidative stress-related enzymes have been also analyzed by two-way ANOVA in relation to different outcome (SR and GR) and controlling for interactions of time from injury (T0, T3, and T6). Most inflammatory parameters were related to the cognitive outcome (Figure 4): IFN-γ (*p* = 0.017), TNF-α (*p* = 0.0033), IL6 (*p* = 0.0170), and IL1β (*p* = 0.0353). In addition, IL6 plasma levels were also associated with differential time from injury (*p* = 0.0342).

Among the oxidative stress, parameters SOD (*p* = 0.0179) and especially GPx (*p* = 2.63e−06) showed an association with time from injury. GPx shows also a possible weak association with cognitive outcome (*p* = 0.065).

**Figure 5:**
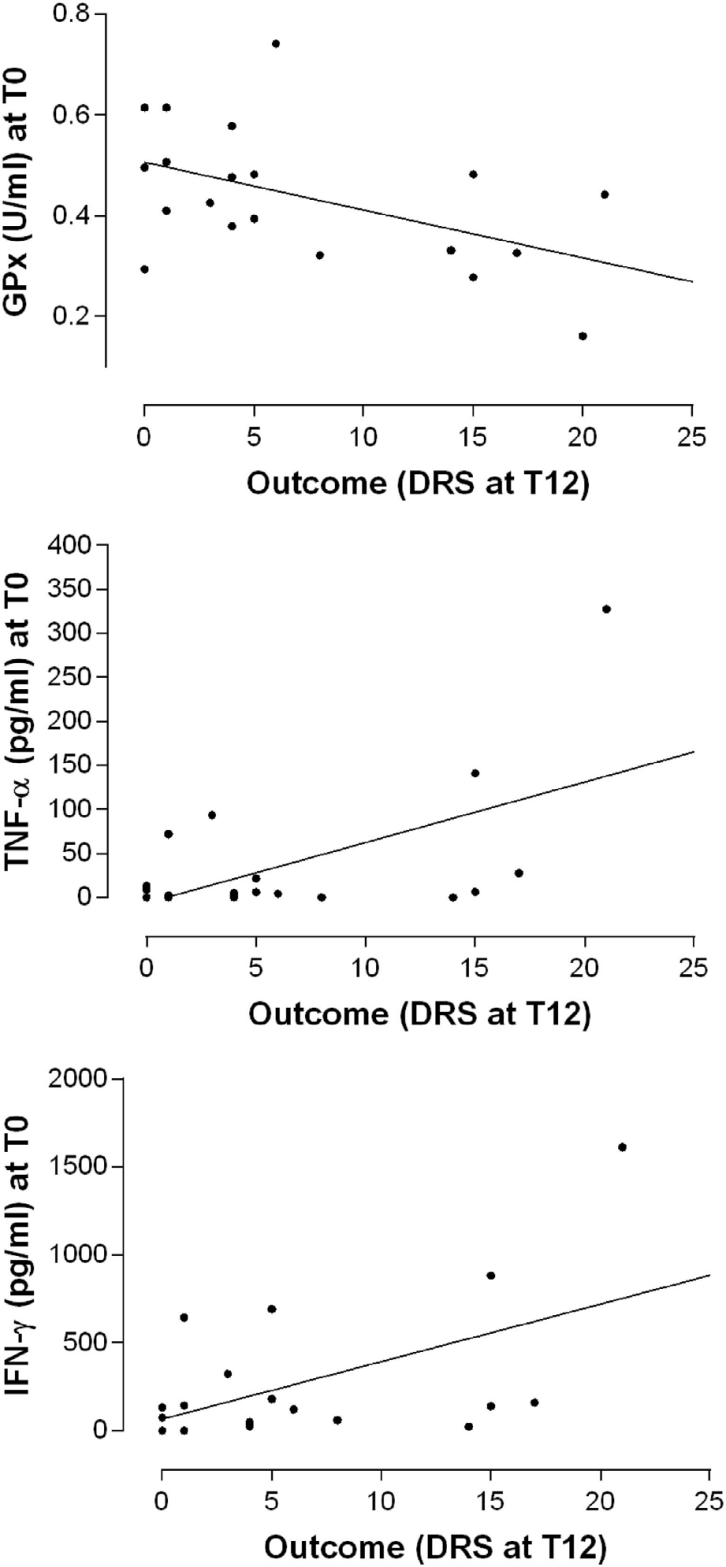
**Relationship between the levels of antioxidant enzyme activities and cytokines at the baseline (T0) and the outcome as measured by the DRS after 12 months**. Simple linear regression models relating GPx (*R*-squared 0.241, *p*-value 0.028), TNF-α (*R*-squared 0.3191, *p*-value 0.0117), and IFN-γ (*R*-squared 0.2735, *p*-value: 0.028) plasma levels at T0 with the overall disability measures at T12.

It is interesting to note that the disability assessed by the DRS score 12 months after the TBI, positively correlated with the T0 levels of IFN-γ (Pearson’s product–moment correlation 0.5229297, *p*-value 0.0216; regression: *R*-squared 0.2735, *p*-value: 0.028) and TNF-α (Pearson’s product–moment correlation 0.5648624; regression: *R*-squared 0.3191, *p*-value 0.0117) while inversely correlated with GPx activity at T0 (Pearson’s product–moment correlation −0.490876, *p*-value 0.02797; Regression: *R*-squared 0.241, *p*-value 0.028) (Figure 5). In other words, high IFN-γ and TNF-α plasma levels and lower GPx activity in the post-acute phase seem to be associated with a poor clinical outcome after the 12-month follow-up.

From T3 to T6, when most of the patients (15/19) could be tested with the neuropsychological battery, high IL6 plasma levels seem to hinder the cognitive functioning (CPI) (Pearson’s product–moment correlation −0.4363128, *p*-value 0.02028) (Table 2). More in detail, higher IL6 plasma levels correlated with lower indexes (SI) of executive functioning (Pearson’s product–moment correlation −0.5119536, *p*-value 0.005354) and attention (Pearson’s product–moment correlation −0.420941, *p*-value 0.0257), but not with the memory index. Similarly, higher plasma levels of IFN-γ and TNF-α correlated with lower executive functioning SI (Pearson’s product–moment correlation −0.4874287, *p*-value 0.008516 and Pearson’s product–moment correlation −0.4687868, *p*-value 0.01186, respectively).

**Table 2:** **Correlation between global cognitive functioning (CPI), synthetic index (SI) of cognitive domains, and plasma level of cytokines and oxidative stress-related enzymes**.

	CPI	ATT	MEM	EXF
GR	0.11933566	−0.07006214	−0.04412505	0.24945769
SOD	−0.01212944	0.06189362	0.16473213	−0.14251454
GPx	−0.14166039	−0.22017577	−0.34377650	0.01092828
CAT	0.24253298	0.33661015	0.16366107	0.22153043
TAA	0.06368209	−0.20511358	−0.12738416	0.20721715
IL1β	−0.08801661	−0.08321005	−0.01759094	−0.17536462
IL6	−0.4363128*	−0.4209410*	−0.2213672	−0.5119536*
IFN-γ	−0.36136382	−0.30798937	−0.14908328	−0.48742874*
TNF-α	−0.36875750	−0.16062906	−0.26462623	−0.46878678*

*All the results at T3 and T6 are considered. The CPI and the SIs (ATT, attention; MEM, memory; EXF, executive functions) are calculated from the neuropsychological tests battery*.

Pearson’s product–moment correlation, *p < 0.05.

Interestingly, all the TBI subjects were APOE ϵ4 carriers (13.6% = ϵ2/4; 86.4% = ϵ3/4), no bias in the recruitment was applicable since patients were genotyped for APOE ϵ4 after the recruitment.

## Discussion

After a severe TBI, the temporal profile of serum inflammatory markers is not completely characterized, while long-term profile of antioxidant related enzyme activity is so far unknown, although it is known that oxidative stress plays a role in TBI (6, 7).

Few studies investigated the cytokines blood levels during periods longer than 7 days (38, 39), and one report (40) investigated cytokines serum levels 3–6 months after the TBI.

While the enzymes involved in oxidative stress are well identified, their imbalance after TBI is not yet determined and published data are mainly derived from animal studies (67–71).

A wide number of enzymes are involved in ROS generation. Superoxide anion and hydroxyl radical are rapidly formed *via* the enzyme NADPH oxidase and contribute to the oxidative stress following TBI (7, 72). Microglial cells are rich in NADPH oxidase and ROS produced by microglia can contribute to neuroinflammation by altering mitochondrial dynamics in astrocytes (73), by amplifying the production of pro-inflammatory cytokines (74), and by exerting direct toxic effects on neurons.

Beyond the NADPH oxidase family, both endothelial and inducible nitric oxide synthase, cytochrome P450, cycloxygenase, lipoxygenase, and XO are involved in mitochondrial dysfunction and excitotoxicity (75).

Superoxide dismutase, GPx, and CAT activities contribute to an increased level of TEAA.

The sample of patients enrolled in this study are not representative of the entire spectrum of TBI severity, since one of the inclusion criteria was a persistent DOC at the time of the enrollment in the study. These patients with a high degree of neurological impairment usually show an uncertain clinical outcome and the pattern of recovery is more often unpredictable.

The majority of the patients had important clinical improvements during the follow-up, but not all with the same rate of recovery. While the cognitive improvement continued for at least 12 months, with the possible exception of attention that seems to worsen slightly between 9 and 12 months after the baseline, most changes in disability and functional independence took place within shorter time periods. Some patients almost completed their improvements in disability and personal independence within 3 months, while others needed longer recovery time up to 9 months. Given these differences in time, it seemed necessary to set a reliable outcome assessment not earlier than 12 months after the enrollment in the study. Moreover, the distinction between post-acute and chronic phase is blurred since significant individual clinical changes occur at different time in different patients. However, it is reasonable to assume as post-acute the clinical phase within 3 months after the TBI. Consequently, T0 falls into the post-acute phase, T3 into an early chronic phase and T6 into the chronic phase.

During the post-acute phase, between 2 and 10 weeks after the TBI (T0, median 32 days), oxidative stress-related enzyme activities and markers of inflammation were abnormally high if compared to previously published data in large populations of unaffected subjects (76–78), even in absence of clinical infections. However, the presence of sub-clinical conditions, such as asymptomatic urinary tract infections or other tissue mild damages, could not be excluded. These factors might be additive, rising further the plasma levels of cytokines. These putative confounding factors cannot be addressed because of the size of patient population.

Oxidative stress parameters and cytokines plasma levels were evaluated in plasma samples at the enrollment in the study (T0), after 3 (T3) and 6 months (T6) after TBI. During the post-acute phase (T0), GPx activity and IL6 levels were significantly higher than during the chronic phase (T6), while, on the contrary, SOD activity increased from the post-acute to the chronic phase. CAT activity did not change over time. The reasons for these differences remain unexplained.

In the chronic phase, between 3 and 6 months after the enrollment in the study, persistent high plasma levels of cytokines appeared to interfere with cognitive functioning, with an exception for memory performance. In fact, elevated IL-6 plasma levels affected both executive functioning and attention, while IFN-γ and TNF-α seem to influence only the executive functions. The association between higher plasma IL6 and lower executive functioning and attention is not specific for brain injured individuals since also in healthy middle-aged (79) and elderly (80) higher levels of IL6 tend to associate with poorer executive functions, but not with memory performances. Anyway, in TBI patients, a persistent over-production of IL6, as well as of IFN-γ and TNF-α, appear to appreciably change the pattern of neuropsychological impairments and time interval of cognitive recovery by slowing and unbalancing improvements of some domains.

Our findings suggest that the assessment of cytokines profile in blood samples may offer a feasible way to investigate long-term processes affecting slowly recovering patients or to identify patients with increased odds of late deterioration.

Oxidative stress and inflammation can affect the TBI clinical outcome, but reports concerning antioxidant responses and inflammatory markers several weeks after the trauma are still scanty. However, this is the time when the patients’ relatives, and the clinical teams, stop worrying only about survival and begin to wonder about long-term disability. Therefore, any information potentially related to cognitive outcome might become clinically relevant.

The inflammatory markers and the oxidative stress-related enzymes have been analyzed for changes related to different cognitive outcome (SR and GR). The inflammatory parameters of the post-acute phase (T0) show an intriguing association with the cognitive outcome (T12): higher levels of inflammatory cytokines (IFN-γ, TNF-α, IL1β, and IL6) at T0 correlated with slower and poorer recovery of cognitive functioning 12 months after TBI. Also, the functional outcome (in terms of chronic disability after 12 months) seems to be influenced by higher post-acute (T0) levels of IFN-γ and TNF-α.

Differently, the GPx plasma activity at T0 inversely correlates with late disability at T12. In other words, higher IFN-γ and TNF-α levels and lower GPx activity at baseline seem to increase the odds of worse DRS scores 12 months later.

Because GPx activity provides a critical protection against lipoperoxide-mediated injury (26), an upregulation during the post-acute phase could be critical in the CNS for the oligodendrocytes. In fact, these cells are particularly vulnerable to oxidative burst because of their low antioxidant enzyme levels (81). A high GPx response might promote repair in damaged white matter tracts or at least provide a line of defense for myelination. This could help to explain the inverse association between the T0 levels of GPx and the outcome at T12.

With different dynamics, the antioxidant response mediated by SOD gradually increased from T0 to T6. Superoxide anions are formed *via* NADPH oxidase, and this enzyme is well represented in microglia cells (72). SOD represents the first mechanism of defense against the superoxide and ROS/RNS levels (82). Because SOD is regulated by its substrate, the persistent and increasing upregulation of this enzyme offers an indirect proof of increased superoxide production. The progressive increase of SOD may be seen as a compensatory mechanism, different from the early rise of activity immediately following the TBI in response to enhanced superoxide anion generation (83) because of BBB damages and peripheral phagocyte cells activation. Overexpression of extracellular SOD in mice has a protective role against brain injury induced by chronic hypoxia (84).

On the contrary, CAT did not significantly change over time. The reasons for this difference remain unexplained.

Presence of the APOE 4 allele may affect inflammatory responses and oxidative stress (85). However, all patients in the present investigation were APOE 4 positive, therefore, differential influence in inflammatory, oxidative markers, and/or clinical outcome cannot be ascribed to this genetic asset.

## Conclusion

Results reported in the present investigation contribute to increase the evidence of long-term persistent inflammatory response after a severe TBI in humans, and, for the first time, provide clues of long-term involvement during the post-acute phase of enzymes related to antioxidant activity.

The chronic overexpression of inflammatory cytokines interfered with cognitive recovery and mainly affected frontal lobe functioning such as the executive functions. Moreover, our findings showed that the over-production of cytokines and the alteration of the redox homeostasis in the post-acute phases of TBI adversely affected the clinical outcome in patients with severe neurological damages. The differences between good recovery and SR groups (identified according to the CPI at T12) also showed that the cognitive outcome might be negatively influenced by higher blood profile of cytokine at the baseline (T0). GPx activity at T0 seemed to influence to some extent the T12 cognitive outcome. However, high data variability was observed in patients of both groups. The differences in demographic and admission characteristics (age, gender, injury details, and CT abnormalities) may be considered confounding factors, but the size of the cohort did not allow us to perform multivariate statistics. Future studies enrolling larger cohorts of patients will be necessary to confirm these preliminary observations and to highlight the relation between the post-acute cytokine profiles and the most predictive factors at admission combined into multivariable prognostic models (86).

So far, this new landscape seems very promising for a deeper understanding of factors adversely affecting the processes of cognitive and motor recovery during the post-acute rehabilitation phase. Inflammatory and antioxidant blood markers might be feasible tools to identify slowly recovering patients and/or those patients with increased odds of late deterioration.

## Author Contributions

FL, SH, and RP designed the study. RP and LS enrolled the subjects and provided the clinical assessments. CS provided the neuropsychological assessments and test analyses. MM, EP, CA, and IC performed the biochemical and immunological measurements. FL, SH, RP, MM, EP, and CS analyzed the data and discussed the results. FL, EP, CS, MM, SH, and RP wrote the manuscript.

## Conflict of Interest Statement

The authors declare that the research was conducted in the absence of any commercial or financial relationships that could be construed as a potential conflict of interest.
